# Iterative Beam Hardening Correction for Multi-Material Objects

**DOI:** 10.1371/journal.pone.0144607

**Published:** 2015-12-10

**Authors:** Yunsong Zhao, Mengfei Li

**Affiliations:** 1 School of Mathematical Sciences, Capital Normal University, Beijing, China; 2 Beijing Higher Institution Engineering Research Center of Testing and Imaging, Beijing, China; 3 Shenzhen ZhongKe TianYue Technology Co., Ltd, Shenzhen, China; 4 Shenzhen Institute of Advanced Technology, Shenzhen, China; Chongqing University, CHINA

## Abstract

In this paper, we propose an iterative beam hardening correction method that is applicable for the case with multiple materials. By assuming that the materials composing scanned object are known and that they are distinguishable by their linear attenuation coefficients at some given energy, the beam hardening correction problem is converted into a nonlinear system problem, which is then solved iteratively. The reconstructed image is the distribution of linear attenuation coefficient of the scanned object at a given energy. So there are no beam hardening artifacts in the image theoretically. The proposed iterative scheme combines an accurate polychromatic forward projection with a linearized backprojection. Both forward projection and backprojection have high degree of parallelism, and are suitable for acceleration on parallel systems. Numerical experiments with both simulated data and real data verifies the validity of the proposed method. The beam hardening artifacts are alleviated effectively. In addition, the proposed method has a good tolerance on the error of the estimated x-ray spectrum.

## Introduction

Beam hardening artifact [1] is one kind of common artifact in x-ray CT (Computed Tomography) images, which usually appears to be cupping artifacts: the outer part of the image has a bigger CT value than the inner part, and streak artifacts that usually appear between any two materials with high absorption coefficients. In Ref. [2], the authors give a detailed description of the appearance of beam hardening artifacts in CT images. Generally, the x-rays emitted from an x-ray source are polychromatic, and the attenuations of materials are energy dependent. Lower energy photons are more easily absorbed than higher energy photons. So the x-rays become harder while propagating through the object. If the energy dependence of the absorption is not taken into account, beam hardening artifact will appear in the reconstructed images. Existence of beam hardening artifact will degrade CT images, or even affects the applicability of the CT images. In order to improve the image quality, beam hardening correction (BHC) methods have been studied even since the invention of the x-ray CT [2, 3].

Existing BHC methods can be classified into hardware methods and software methods. In hardware methods, thin metal plates are placed between the x-ray source and the scanned object to narrow the broad source spectrum [4]. Hardware methods are easy to implement, but can only reduce the beam hardening artifacts in the reconstructed image to some degree; and the signal to noise ratio of the scanned data will decrease, as the filters absorb parts of the x-ray photons.

Software methods can further be classified into: linearization methods, dual energy methods, and iterative methods.

Linearization methods construct a map between the polychromatic projections and the linear projections of the scanned object by some methods [2, 3, 5, 6]. With this map, linear projections of the scanned object are calculated from its polychromatic projections. Next CT images of the scanned object is reconstructed from its linear projections with traditional methods, such as the FBP (Filtered Backprojection) [7] or SART (Simultaneous algebraic reconstruction technique) [8] methods. Polynomial fitting method is one of the linearization methods that are commonly used. This method assumes that the map is a polynomial, whose coefficients are usually obtained by scanning some standard phantoms [5]. The precision of the map is of vital importance, the errors in the map may introduce systematic errors in the reconstructed images.

For the dual energy methods [9–12], the linear attenuation coefficient of the scanned object is decomposed into the photoelectric component and the Compton scatter component or two basis material components. In order to determine the images of the two components, two scans at different source voltages are required. After the images are determined by specific dual energy reconstruction methods, the image of the linear attenuation coefficient of the scanned object can be estimated by a linear combination of the reconstructed two images.

Iterative methods incorporate the polychromatism of the x-ray spectrum and the dependence of the attenuation with the x-ray energy into image reconstruction models [13–15]. The models approximate the physical process better, and usually get better correction results. In Ref. [13], the authors propose a beam hardening correction method which requires no prior knowledge of the scanned object or the x-ray beam. The method introduces two parameters in the forward projection process to compensate the hardening of the x-ray beam. It works fine for single material object. But for object with multiple materials, it is hard to select proper parameters, and often results in over corrected or under corrected images. In Ref. [14], the authors constructed a cost function that measures the differences between the estimated polychromatic projections and the real ones. By minimizing the cost function, the attenuation coefficients, the x-ray spectrum, and the density of each material in the scanned object are estimated at the same time. As the cost function defined is not convex, the solution is not unique. The initial values are important to obtain the reasonable solution. In Ref. [15], Krumm etal propose an iterative BHC method which also does not require prior knowledge on the spectrum or material attenuations. As image segmentation technique is used in the method, the method can successfully suppress beam hardening artifacts in the images when an adequate segmentation can be performed based on the uncorrected reconstruction. The large calculation amount is the main reason that limits their applications. While with the rapid development of parallel computing hardware, such as the GPUs (Graphics Processing Units), iterative methods get more and more researchers’ attention.

In this paper, we propose an iterative BHC method that is applicable for the case with multiple materials. First, the BHC problem is converted into a nonlinear system problem by introducing some assumptions on the scanned object, i.e., the materials composing scanned object are known and they are distinguishable by their linear attenuation coefficients at some given energy. Then the nonlinear system is solved iteratively. The reconstructed image, i.e., the solution of the nonlinear system, is the distribution of linear attenuation coefficient of the scanned object at a given energy. So there are no beam hardening artifacts in the image theoretically. The iterative scheme for solving the nonlinear system includes two procedures: forward projection and backprojection. In forward projection, both the spectrum of the x-ray and the linear attenuation coefficients of the materials are incorporated in the calculation of the estimated polychromatic projections of the estimated image. In backprojection, the weighted residuals, i.e., the weighted differences between the real projections and the estimated projections, are added back on the estimated image, where the weights are selected according to both the intersection length of the x-ray path and the pixels and the estimated values of the pixels. The method is verified with both simulated data and real data. The reconstruction results show that the proposed method can alleviate the beam hardening artifacts effectively. In addition, the proposed method has a good tolerance on the error of the estimated x-ray spectrum. Besides, the proposed method is suitable for acceleration on GPUs or other parallel systems.

## Model and Method

In this section, we fist derive the nonlinear system that models the beam hardening correction problem; then give an iterative scheme that solves the nonlinear system. Some numerical implementation details are also presented.

### Model

It is known that, when omitting the scattered photons, the relation between the polychromatic projections and the linear attenuation coefficient of the scanned object is as follows [11]:

$$p\left(L\right)=-\text{log}\int_{0}^{E_{MAX}}S\left(E\right)e^{-\int_{L}\mu \left(E,x\right)dl}dE,\;L\in L$$ (1)

where *p*(*L*) indicates the polychromatic projection along the x-ray path L∈L, and L is the set of x-ray paths. *μ*(*E*, ***x***) indicates the energy-dependent spatial distribution of the linear attenuation coefficient. *E* is the photon energy. *S*(*E*) is the normalized x-ray spectrum, which presents compositive effect of the emission spectrum of the x-ray tube, the material and thickness of the detector scintillator, the material and thickness of the filter, etc. The CT imaging problem is to calculate the distribution of the linear attenuation coefficient *μ*(*E*, ***x***) from obtained polychromatic projections *p*(*L*) along different x-ray paths L∈L.

Generally, (1) is an underdetermined system, whose solution is not unique. In order to obtain the solution of that represents the real attenuation distribution of the scanned object, prior information of the scanned object is usually incorporated in the image reconstruction model. Ref. [10] and Ref. [16] assumed volume conservation that the volume of a mixture equals to the sum of volumes of its constituent parts, and assumed that there are at most 3 different materials at each point of the mixture. Ref. [17] used air, water, bone, and iron as references, other materials’ attenuation coefficients are interpolated from that of the reference materials.

In this paper, it is assumed that: 1) the materials composing the scanned object are known; 2) the density of each material may change a little from its nominal density, but the change cannot be too much; 3) each point of the scanned object is occupied by only one material. It should be noted that some objects may not satisfy the assumptions mentioned above. But for many detection tasks, the assumptions above are reasonable, especially for nondestructive test of workpieces.

Let *K* be the number of materials composing the scanned object, and label different materials with 1, 2,…, *K*. Let Ξ = {1,2,…,*K*} be the set of the labels. Let $\mu_{m}^{k}\left(E\right)$ denote the mass attenuation coefficient of the *k*-th material, and *ρ*(***x***) denote the density distribution of the scanned object.

Define the characteristic function of the *k*-th material *χ*(***x***, *k*), whose value is 1 if the point ***x*** is occupied with the *k*-th material, and 0 otherwise. Then the linear attenuation coefficient of the scanned object *μ*(*E*, ***x***) can be written as

$$\mu \left(E,x\right)=\sum_{k=1}^{K}\chi \left(x,k\right)\mu_{m}^{k}\left(E\right)\rho \left(x\right)$$ (2)

We know that different materials have different attenuation curves, i.e., the curve of *μ*(*E*, ***x***) with the first variable *E*. Under the assumptions mentioned earlier, there exists an energy value *E*_0_, at which the *K* materials composing the scanned object have different linear attention coefficients. So *μ*(*E*_0_, ***x***) can be used to distinguish materials. Let

$$\Theta_{E_{0}}\left(\mu \right):R\to \Xi$$ (3)

be the function that maps the linear attenuation coefficient to its corresponding material label. Define

$$\hat{\chi }\left(\mu ,k\right)=\{\begin{array}{cc} 1, & \Theta_{E_{0}}\left(\mu \right)=k\\ 0, & otherwise \end{array}$$ (4)

Then we have

$$\hat{\chi }\left(\mu_{E_{0}}\left(x\right),k\right)=\chi \left(x,k\right)$$ (5)

Please note that such *E*_0_ is easy to select. Actually, any *E* such that the *K* attenuation curves do not intersect can be used as *E*_0_.

Substitute (5) and the identity $\mu_{m}^{k}\left(E\right)=\frac{\mu_{m}^{k}\left(E\right)}{\mu_{m}^{k}\left(E_{0}\right)}\mu_{m}^{k}\left(E_{0}\right)$ into (2), we have

$$\mu \left(E,x\right)=\sum_{k=1}^{K}\hat{\chi }\left(\mu_{E_{0}}\left(x\right),k\right)\frac{\mu_{m}^{k}\left(E\right)}{\mu_{m}^{k}\left(E_{0}\right)}\mu_{m}^{k}\left(E_{0}\right)\rho \left(x\right)=\sum_{k=1}^{K}\hat{\chi }\left(\mu_{E_{0}}\left(x\right),k\right)\frac{\mu_{m}^{k}\left(E\right)}{\mu_{m}^{k}\left(E_{0}\right)}\mu \left(E_{0},x\right)$$ (6)

where $\mu \left(E_{0},x\right)=\sum_{k=1}^{K}\chi \left(x,k\right)\rho \left(x\right)\mu_{m}^{k}\left(E_{0}\right)$. For simplicity, define ${\hat{\mu }}_{m}^{k}\left(E\right)=\frac{\mu_{m}^{k}\left(E\right)}{\mu_{m}^{k}\left(E_{0}\right)}$, then we have

$$\mu \left(E,x\right)=\sum_{k=1}^{K}\hat{\chi }\left(\mu_{E_{0}}\left(x\right),k\right){\hat{\mu }}_{m}^{k}\left(E\right)\mu \left(E_{0},x\right)$$ (7)

Substitute (7) into (1), we have

$$p\left(L\right)=-\text{log}\int S\left(E\right)e^{-\sum_{k=1}^{K}{\hat{\mu }}_{m}^{k}\left(E\right)\int \;\hat{\chi }\left(\mu_{E_{0}}\left(x\right),k\right)\mu_{E_{0}}\left(x\right)dl}dE$$ (8)

Now we discretize (8). Let ***S*** = (*S*_1_, *S*_2_,⋯,*S*_*N*_) be the sampling values of *S*(*E*), with sampling interval *δ*, ${\hat{\mu }}_{m}^{k,n}$ be the sampling value of ${\hat{\mu }}_{m}^{k}\left(E\right)$ corresponding to *S*_*n*_. Let ***μ*** = (*μ*_1_, *μ*_2_,⋯,*μ*_*J*_) be the discrete image of *μ*(*E*_0_, ***x***). We obtain the discrete form of polychromatic projections

$$p_{i}=-\text{log}\sum_{n=1}^{N}S_{n}\delta e^{-\sum_{k=1}^{K}{\hat{\mu }}_{m}^{k,n}\sum_{j=1}^{J}R_{ij}\hat{\chi }\left(\mu_{j},k\right)\mu_{j}}\;,\;i\in I$$ (9)

where I is the index set of x-ray paths, whose cardinality is *I*, *R*_*ij*_ represents the contribution of the *j*-th pixel of the image ***μ*** to the polychromatic projection *p*_*i*_ along the *i*-th x-ray path.

After discretization, the image reconstruction problem is converted to solving the nonlinear system (9) to obtain the discrete image ***μ*** from a series of polychromatic projections $p_{i},i\in I$. Please note that the image ***μ*** is the distribution of the linear attenuation coefficient of the scanned object at the given energy *E*_0_. So there are no beam hardening artifacts in the image theoretically. In the following subsection, we propose an iterative method for solving the nonlinear system. As there are no beam hardening artifacts in the reconstructed image, the proposed image reconstruction method can be regarded as a BHC method.

### Method

There are many methods for solving the problem (12), such as the classic Newton method [18]. In Ref. [9], we proposed an iterative method, named E-ART (Extended Algebraic Reconstruction Technique), for solving nonlinear system problems. The E-ART method is a row-action method, and suitable for large scale problems. In this paper, the E-ART method is employed for solving the BHC problem.

The E-ART method combines an accurate polychromatic forward projection with a linearized backprojection. The accurate polychromatic forward projection step guarantees that the polychromatic projections of the reconstructed images converge to the measured projections; the linearization in backprojection simplifies the allocation of the residuals.

Suppose that after *r* iterations, we have estimated image ***μ*** ^(*r*)^. Now we are going to use the *i*-th polychromatic projection *p*_*i*_ to update the estimated image. The iterative scheme of E-ART is as follows

$$\mu^{\left(r+1\right)}=\mu^{\left(r\right)}+\frac{p_{i}-p_{i}\left(\mu^{\left(r\right)}\right)}{\left|A_{i}\right|}\frac{A_{i}}{\left|A_{i}\right|}$$ (10)

where ***A***_*i*_ is the gradient of *p*_*i*_(***μ***) at ***μ*** = ***μ*** ^(*r*)^, and |*A*_*i*_| is the L2 norm of *A*_*i*_.

Let ***A***_*i*_ = (*a*_*i*1_, *a*_*i*2_,⋯,*a*_*iJ*_)^*τ*^, where *τ* denotes the transpose of a vector, then we have

$$a_{ij}=-\frac{\sum_{n=1}^{N}S_{n}\delta e^{-\sum_{k=1}^{K}{\hat{\mu }}_{m}^{k,n}\sum_{l=1}^{J}R_{il}\hat{\chi }\left(\mu_{l}^{\left(r\right)},k\right)\mu_{l}^{\left(r\right)}}\left(\sum_{k=1}^{K}{\hat{\mu }}_{m}^{k,n}R_{ij}\left({\hat{\chi }}^{\prime }\left(\mu_{j}^{\left(r\right)},k\right)\mu_{j}^{\left(r\right)}+\hat{\chi }\left(\mu_{j}^{\left(r\right)},k\right)\right)\right)}{\sum_{n=1}^{N}S_{n}\delta e^{-\sum_{k=1}^{K}{\hat{\mu }}_{m}^{k,n}\sum_{l=1}^{J}R_{il}\hat{\chi }\left(\mu_{l}^{\left(r\right)},k\right)\mu_{l}^{\left(r\right)}}}$$ (11)

where ${\hat{\chi }}^{\prime }\left(\mu_{j},k\right)$ denotes the partial derivative of $\hat{\chi }\left(\mu_{j},k\right)$ on the first variable. Under the assumptions mentioned earlier, $\hat{\chi }\left(\mu_{j},k\right)$ is piece-wise constant, and has discontinuous points. So here ${\hat{\chi }}^{\prime }\left(\mu_{j},k\right)$ should be regarded as the generalized partial derivative of $\hat{\chi }\left(\mu_{j},k\right)$. In numerical computation, we may use Gaussian function with small deviation to approximate ${\hat{\chi }}^{\prime }\left(\mu_{j},k\right)$. While from our numerical experiments, we find that just let ${\hat{\chi }}^{\prime }\left(\mu_{j},k\right)=0$ will also get satisfactory results.

We summarize the implementation steps of the iteration scheme as follows.

**Step 1**. Assign ***μ*** ^(0)^ with some initial values.

**Step 2**. Suppose the estimations ***μ*** ^(*r*)^ are known after *r*(≥0) iterations. For a given x-ray path along which the polychromatic projection is *p*_*i*_, calculate *p*_*i*_(***μ*** ^(*r*)^), and ***A***_*i*_ by (9) and (11).

**Step 3**. Calculate ***μ*** ^(*r*+1)^ by (10).

**Step 4**. Stop if the convergence criterion is satisfied, otherwise turn to Step 2.

In numerical experiments, the iteration is stopped when the relative change of the two adjacent reconstructed images is smaller than some given value or the maximum iteration is reached. The measurement of the relative change used in our experiments is

$$e\left(r\right)=\frac{{\left\|\mu^{\left(r\right)}-\mu^{\left(r-1\right)}\right\|}_{2}^{2}}{{\left\|\mu^{\left(r-1\right)}\right\|}_{2}^{2}}$$ (12)

Where ‖⋅‖_2_ denotes the *L*_2_ norm of a vector. The threshold for *e*(*r*) used in the experiments is 10^−4^ and the maximum number of iteration is 100. The initial value used in our experiments is zero, that is ***μ*** ^(0)^ = ***0***. The value of $\hat{\chi }\left(\mu_{j}^{\left(r\right)},k\right)$ is obtained by comparing the value of $\mu_{j}^{\left(r\right)}$ with preselected thresholds, which are determined by the knowledge of the materials composing the object.

Note that in the calculation of both *p*_*i*_ and *a*_*ij*_, we need the value of $\sum_{j}^{J}R_{ij}\hat{\chi }\left(\mu_{j}^{\left(r\right)},k\right)\mu_{j}^{r}$, which is the linear forward projection of $\mu_{j}^{r}$, with weight $\hat{\chi }\left(\mu_{j}^{\left(r\right)},k\right)$. The values for different *k* can be obtained in one forward projection process. In numerical implementation, the ray casting method and the pixel driven method can be used in forward projection and backprojection, which are suitable for acceleration on GPUs (graphic processing units) or other parallel systems. For the detailed information of the two techniques, please refer to [19].

## Numerical Experiments

In this section we perform numerical experiments with both simulated data and real data to validate our method. For simplicity, the numerical experiments are restricted to fan beam CT. Generalization to cone-beam CT is straightforward.

For comparison, we have also implemented the iterative BHC method proposed by Brabant etal in Ref. [13]. For simplicity, we call it Brabant method in the rest of the paper. The Brabant method compensates the hardening of x-ray beam by introducing two parameters in the forward projection process of the iteration. In order to obtain the approximately optimal parameters for the method, we reconstruct images with different parameters that are distributed uniformly in a large interval, and then choose the parameters that have the best correction result. The interval tested is [10^−4^, 5×10^−4^]×[2.0, 3.6], the sampling steps for the two parameters are 2×10^−5^ and 0.2, respectively. With these parameters, both over corrected and under corrected results are obtained. So the optimal parameters should be included in the tested interval.

### Simulated data experiments

**Fig 1:**
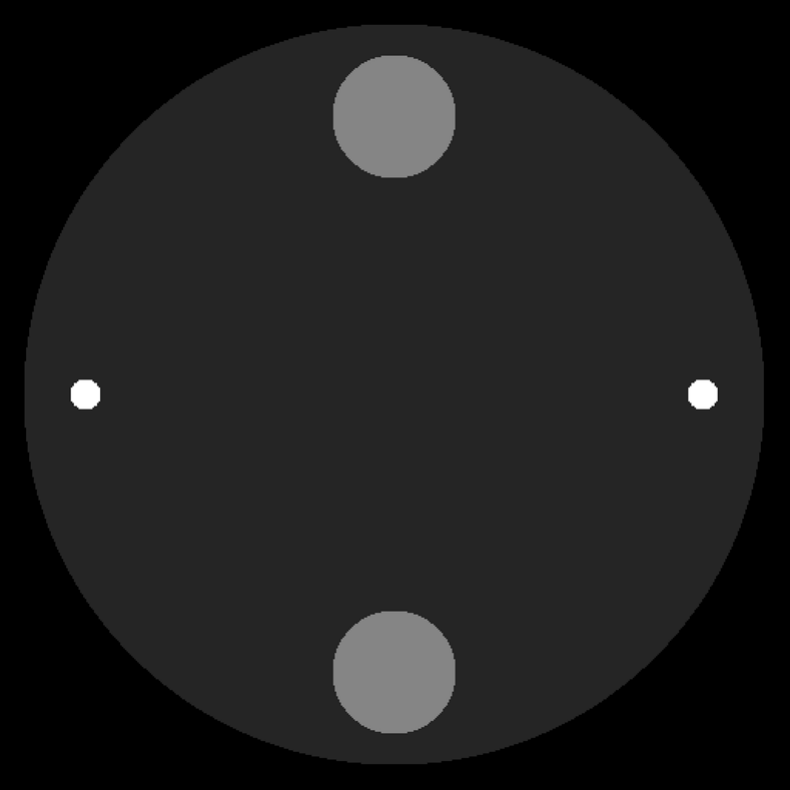
Numerical phantom for simulation.

The numerical phantom used in the simulation is shown in Fig 1, which includes three materials: water, bone, and titanium. The biggest disk is filled with water, and its diameter is 240 mm; the two bigger ones of the other four disks are filled with bone, and their diameters are both 40 mm; the smallest two disks are filled with titanium and their diameters are 10 mm.

**Fig 2:**
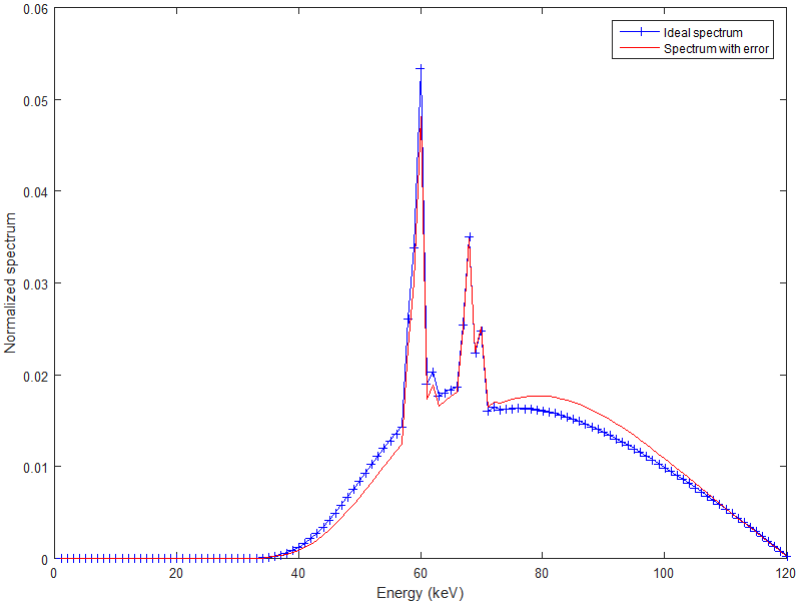
The simulated x-ray spectrum (blue line) and the error-included spectrum (red line).

The polychromatic x-ray spectrum is simulated with the software SpectrumGUI, an open source x-ray spectra simulator (see http://spectrumgui.sourceforge.net). We have simulated the x-ray spectrum of GE Maxiray 125 x-ray tube with tube voltage 120 kV and 1 mm copper filter. The distribution of the x-ray spectrum is shown in Fig 2 (the blue one). The mean energy of the spectrum is 75.29 keV.

The mass attenuation coefficients used in the experiments are retrieved from the National Institute of Standard Technology (NIST) tables of x-ray mass attenuation coefficient [20].

The parameters of the scanning configuration are set as follows: the distance from the x-ray source to the rotation center is 1000 mm, and the distance from the x-ray source to the linear detector is 1200 mm. The linear detector is composed of 512 detector cells, and the length of each cell is 0.6 mm. The cell size is selected to accommodate a proper field of view. With the above parameters, the diameter of the field of view is 256 mm. 720 projections are simulated on one full turn of the turntable.

We have also simulated noisy data to verify the noise performance of the proposed iterative BHC method. The Poisson noise data is simulated corresponding to emission flux of 10^5^ photons per measurement.

**Fig 3:**
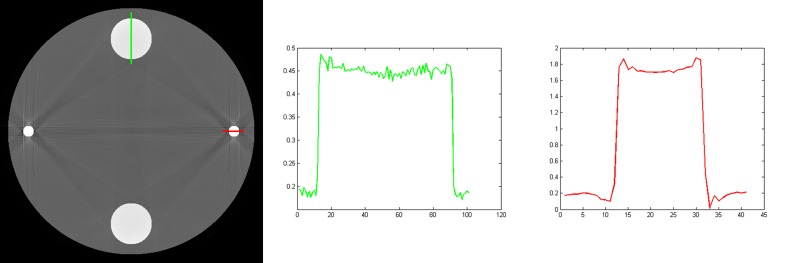
The results reconstructed with the SART method. The left column is the reconstructed image, and the right two columns are the profiles along the lines marked in the reconstructed image. The display window for the reconstructed image is [0, 0.5].

First we reconstruct image with traditional single energy reconstruction method. In our experiments, the SART method is used. The left image shown in Fig 3 is the reconstruction result. The display window for this image is [0, 0.5]. From the reconstructed image, we can see obvious streak artifacts between bone and titanium materials. The right two images in Fig 3 are the profiles along the lines marked in the left image. We can see that the curves are concave because of beam hardening.

It should be noted that the vertical streaks tangent to the titanium materials shown in the reconstructed image are caused by the geometric singularity of the numerical phantom. The reason that the streaks are shown along vertical direction is that among all lines tangent to the titanium materials, the vertical lines have the shortest intersection lengths with the water material. The titanium materials have more effect on the polychromatic projections along these lines than that along other lines.

Now we reconstruct images with our method and the Brabant method. As is mentioned earlier that our method reconstruct linear attenuation coefficient of the scanned object at a given energy *E*_0_. The energy selected in our experiments is *E*_0_ = 50 keV. At this energy, the linear attenuation coefficients for water, bone and titanium are 0.236, 0.837, and 5.518 with unit cm^−1^, respectively.

**Fig 4:**
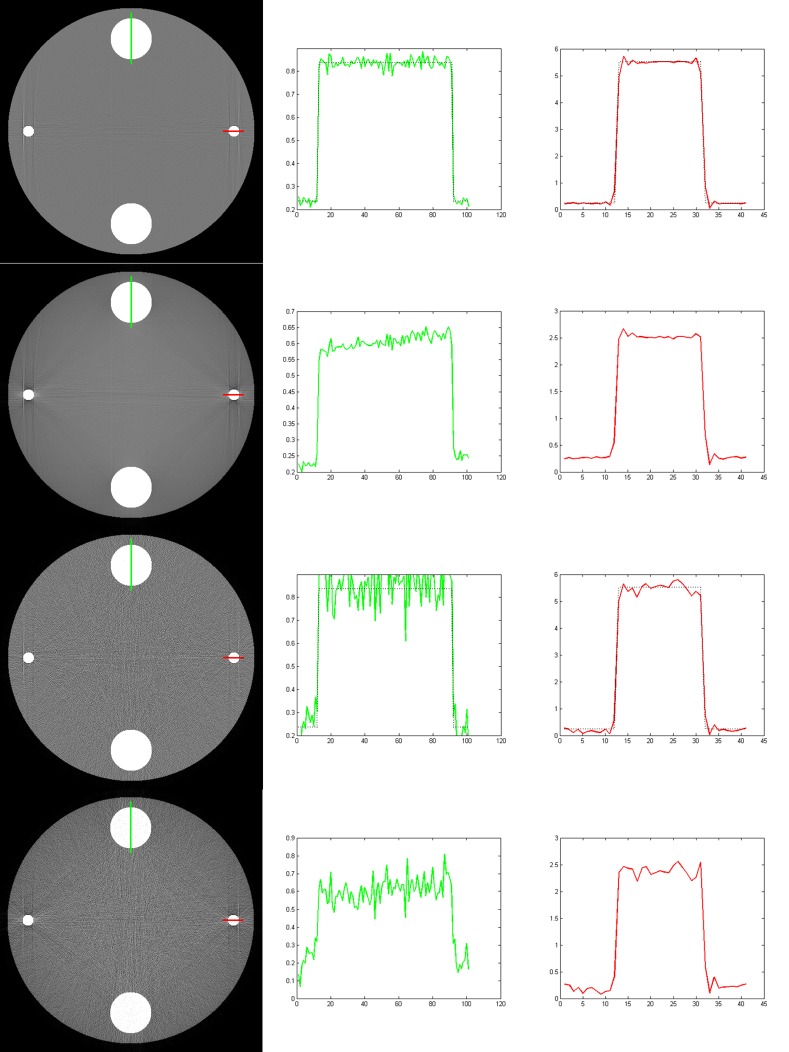
The results reconstructed with our method (the first and the third rows) and the Brabant method (the second and the fourth rows) from noise free data (the first and the second rows) and noisy data (the third and the fourth rows). The left column is the reconstructed images, and the right two columns are the profiles along the lines marked in the reconstructed images. The display window for the reconstructed images is [0, 0.5].

The results reconstructed with our method and the Brabant method are shown in Fig 4, where the first and the third rows are the images reconstructed with our method, the second and the fourth rows are the images reconstructed with the Brabant method. The images in first and the second rows are reconstructed from noise free data, but in the third and the fourth rows are reconstructed from noisy data. The middle and the right column are the profiles along the lines marked in the corresponding reconstructed images in the left row. The display window for the reconstructed images is [0, 0.5]. The iteration numbers of our method for noise free and noisy data are 6 and 10, respectively. While for the Brabant method, the maximum iteration number is reached for both noise free and noisy data. Actually, we find that the relative change of the Brabant method oscillates around 10^−3^ after a few iterations. It can be seen that both method have effects on alleviating cupping artifacts and streak artifacts. In the images reconstructed with the Brabant method, there are slight streaks near the titanium disks. While in the images reconstructed with our method, there are no obvious streak artifacts. In addition, we can see from the profiles that the Brabant method corrects the titanium disks well, but the bone disks are over corrected. While our method corrects the beam hardening artifacts for all materials well. The reconstructed linear attenuation coefficients (the solid lines) are close to their theoretical ones (the dashed lines).

Again, note that there still are singularity artifacts (vertical lines tangent to the titanium materials) in the reconstructed images. Regularization constraints, such as TV minimization, on the images may alleviate the singularity artifacts. But as our main purpose is to verify the validity of our BHC method, no regularization is applied.

The images reconstructed with the Brabant method are slightly inferior to that reconstructed with our method. But it should be pointed out that the Brabant method uses less prior knowledge. So Brabant method can be used in the case that the composition of the object are complex, such as rock cores.

The x-ray spectrum for a real CT system is usually obtained from the manual of the x-ray generator, or estimated by experiments. So there are inevitable errors between the estimated spectrum and the real one. In the next experiment, we test the sensitivity of our method to the errors of the estimated x-ray spectrum.

In order to introduce errors in the ideal spectrum, the detector response is considered. The error-included spectrum is obtained by multiplying the ideal spectrum with an efficiency function $1-0.25\;\text{sin}\left(2\pi \frac{E}{E_{max}}\right)$. The red curve shown in Fig 2 is the spectrum with errors.

**Fig 5:**
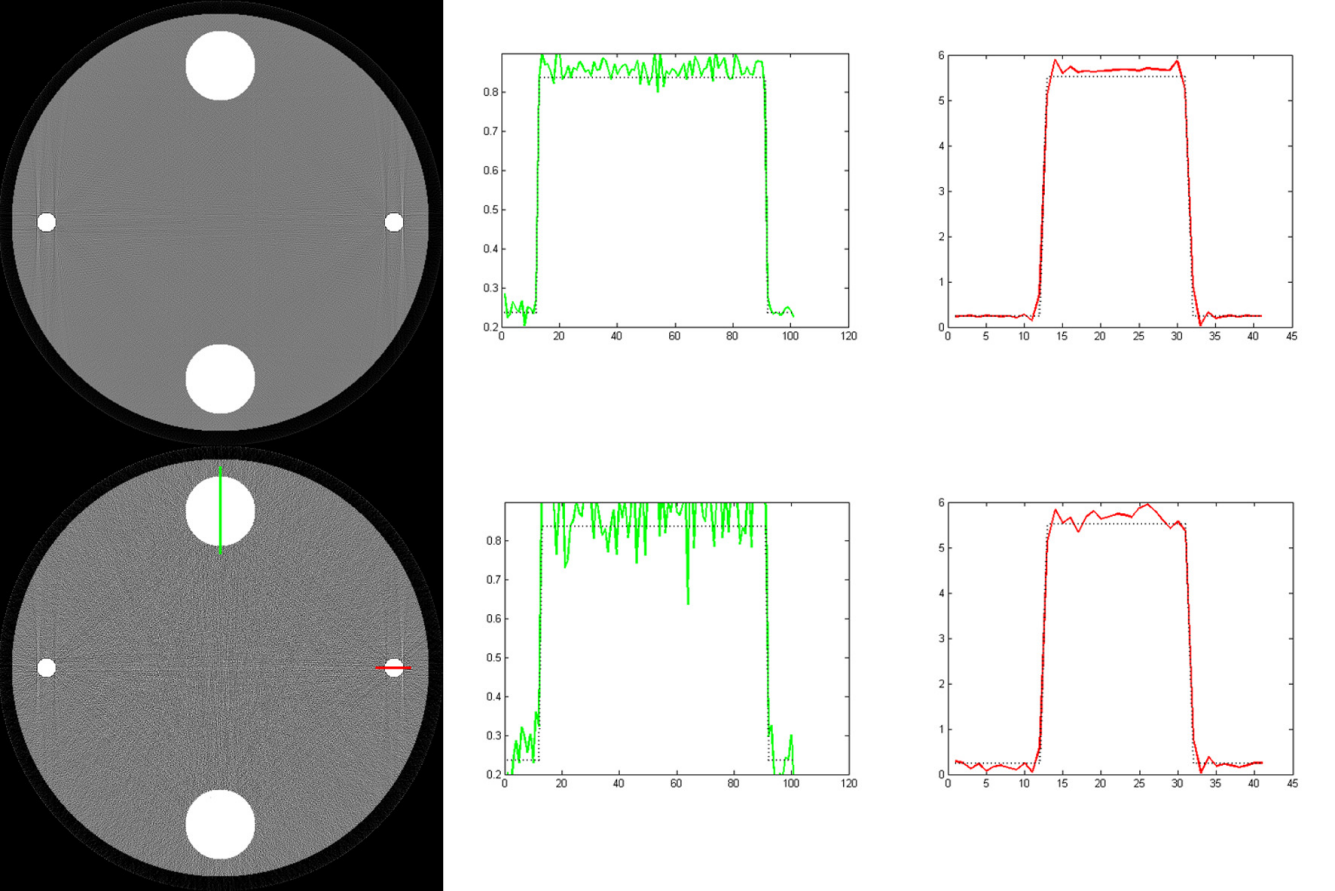
The results reconstructed with our method from noise free data (the first row) and noisy data (the second row) using error-included x-ray spectrum. The left column is the reconstructed images, and the right two columns are the profiles along the lines marked in the reconstructed images. The display window for the reconstructed images is [0, 0.5].

With this error-included spectrum, we reconstruct images from noise free and noisy projections. The results are shown in Fig 5. The iteration numbers for noise free and noisy data are 7 and 13, respectively. Although there are obvious differences between the ideal spectrum and the one with errors (See Fig 2), there are no visible beam hardening artifacts in the reconstructed image, which means that our BHC method has a good tolerance on the errors in the estimated x-ray spectrum.

For quantitative analysis, we have calculated the MSE (Mean Squared Error) and the NMSD (Normalized Mean Square Distance) of the images reconstructed with our method shown in Figs 4 and 5. The errors and distances for the results reconstructed with the Brabant method are not given, as the reconstructed values with the Brabant method do not have a definite meaning, so we have no references for calculating the values. Table 1 shows the values of the two measures. We can see that the differences of the two measures between the images reconstructed from noise free projections with ideal spectrum and with error-included spectrum are very small, which again shows that our BHC method has a good tolerance on the errors in the estimated x-ray spectrum.

**Table 1:** MSE and NMSD of the images shown in Figs 4 and 5.

	MSE	NMSD
Ideal spectrum, noise free projections	0.000911	0.098154
Ideal spectrum, noisy projections	0.002560	0.164511
Error-included spectrum, noise free projections	0.001132	0.123326
Error-included spectrum, noisy projections	0.002648	0.176165

### Real data experiments

**Fig 6:**
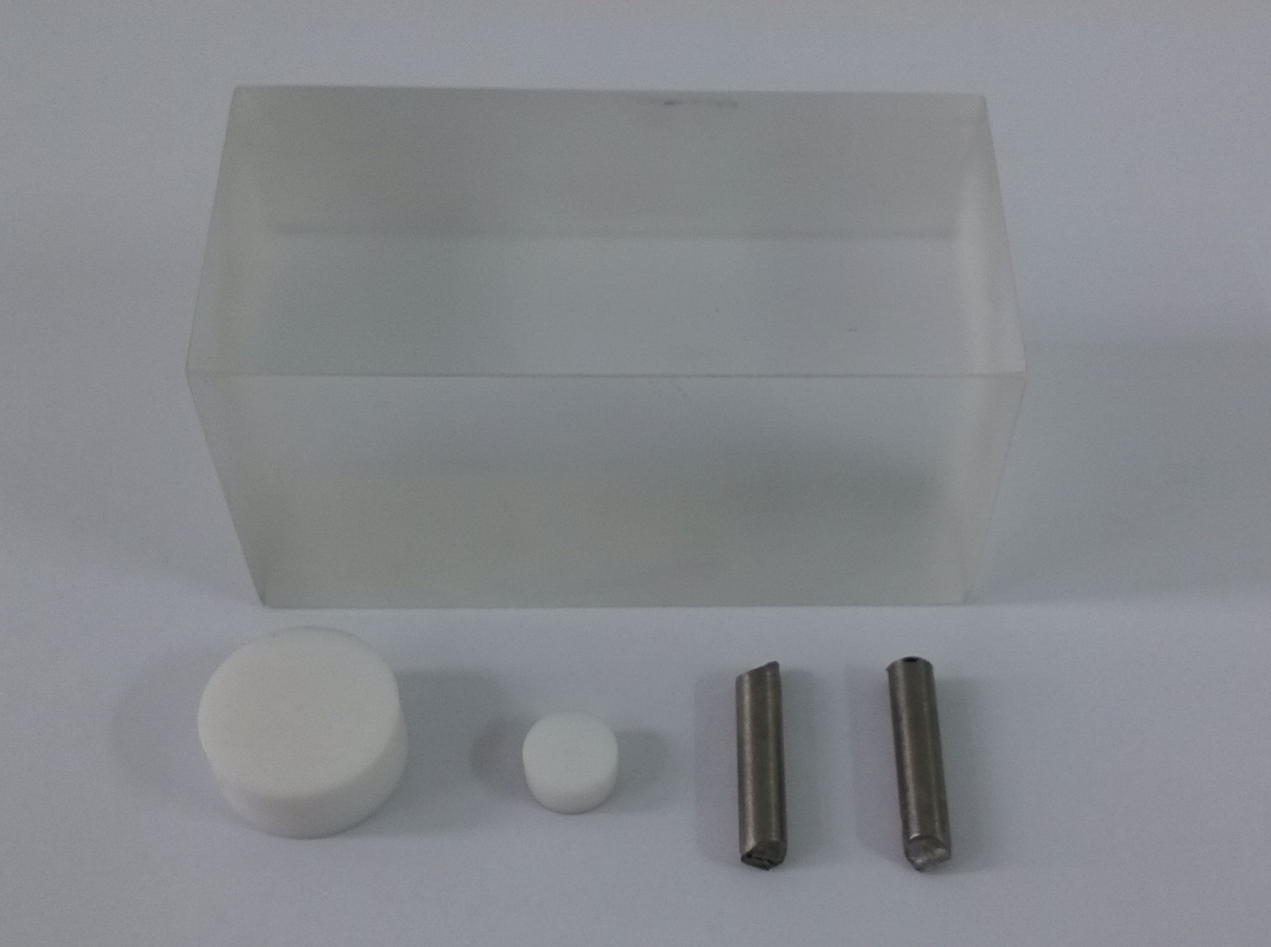
The photograph of the scanned phantom.

In this subsection, we reconstruct images from real data. The phantom used includes: 1) a cuboid water equivalent phantom whose cross section is 40x80 mm^2^; 2) two cylindrical bone equivalent phantoms with diameters 20 mm and 10 mm respectively; 3) two cylindrical titanium alloy phantoms with diameters 5 mm. Fig 6 shows the photograph of the phantom.

The data are scanned with an experimental CT system in our lab, equipped with an YXLON.TU/450-D03 x-ray source and a NTB W2-320 detector whose cell size is 0.083 mm.

The parameters of the scanning configuration are set as follows: the distance from the x-ray source to the rotation center is 530 mm, and the distance from the x-ray source to the linear detector is 810 mm. The scan voltage and current are 120 kV and 2 mA, respectively. The exposure time for each projection is 30 ms. 720 projections are acquired on one full turn of the turntable.

**Fig 7:**
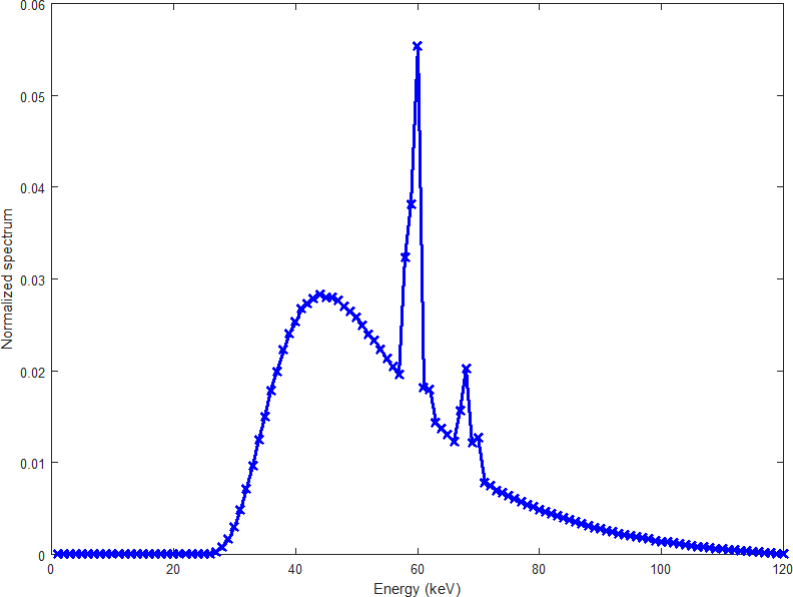
The estimated spectrum of the x-ray source with voltage 120 kV.

The spectrum of the x-ray source is estimated with the EM method from transmitted data [21]. The estimation result is shown in Fig 7. It should be noted that the estimated spectrum is a compositive spectrum, including the emitted spectrum of the x-ray source and the response of the detector, and etc.

The energy *E*_0_ selected here is also 50 keV. The attenuation of pure titanium is used in our experiments disregarding other materials composing the titanium alloy.

**Fig 8:**
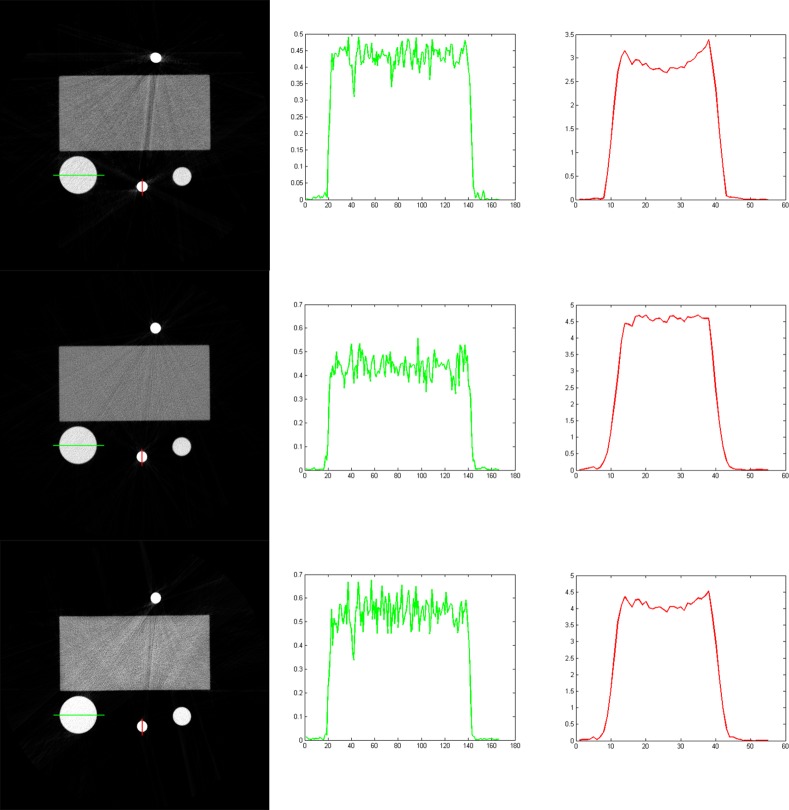
The reconstructed results with the SART method (the top row), our method (the middle row), and the Brabant method (the bottom row). The left column is the reconstructed images, and the right two columns are the profiles along the lines marked in the reconstructed images. The display window for the reconstructed images is [0, 0.5].

Fig 8 shows the results reconstructed with the SART method (the top row), our method (the middle row), and the Brabant method (the bottom row). The display window for the reconstructed images is [0, 0.5]. Obvious cupping artifacts are shown in the profiles of the result reconstructed with the SART method, and that there are streak artifacts between the two titanium alloys. The Brabant method corrects the cupping artifacts of the bone material, and alleviates cupping artifacts of the titanium material, but not quite well; while our method corrects the cupping artifacts for all the materials.

From the profiles in Fig 8, we can see that, with our method, the reconstructed linear attenuation coefficient of the titanium alloy is about 4.6 cm^−1^. There appears some deviation between the reconstructed value and the theoretical one (the theoretical value is 5.518 cm^−1^). We guess it is caused by the impurity of titanium.

It should be noted that there are slight radial artifacts between high absorption materials in the images reconstructed with our method. We analyze that it is caused by the quantum noises in the scanned data.

## Conclusion

In this paper, an iterative BHC method is proposed which reconstructs the linear attenuation coefficients of the scanned object at a given x-ray energy from obtained polychromatic projections. Numerical experiments with both simulated data and real data verify its validity. The beam hardening artifacts in the CT images are alleviated effectively with the proposed method.

For a real system, scatter is inevitable, especially in the context of cone beam CT. Existence of scatter photons in the scanned data will influence the performance of the proposed method. One straight forward method to reduce the effect of scatter is to add a scatter correction process before BHC, which is another important research area in x-ray CT imaging and there are already many effective methods proposed in literals. Please refer to Ref. [22] and the references therein for detailed information.

In the images reconstructed from real data with our method, there are slight radical artifacts remained. The radical artifacts are believed to be caused by the quantum noises in the scanned data. The elimination of the radical artifact will be studied in the future.
